# Biomedical Applications of Antiviral Nanohybrid Materials Relating to the COVID-19 Pandemic and Other Viral Crises

**DOI:** 10.3390/polym13162833

**Published:** 2021-08-23

**Authors:** Shahin Homaeigohar, Qiqi Liu, Danial Kordbacheh

**Affiliations:** 1 School of Science and Engineering, University of Dundee, Dundee DD1 4HN, UK; 2436650@dundee.ac.uk; 2 School of Biomedical Engineering, Southern Medical University, Guangzhou 510515, China; Qiqi_liu99@163.com

**Keywords:** nanohybrid, nanocomposite, COVID-19, biomedical application

## Abstract

The COVID-19 pandemic has driven a global research to uncover novel, effective therapeutical and diagnosis approaches. In addition, control of spread of infection has been targeted through development of preventive tools and measures. In this regard, nanomaterials, particularly, those combining two or even several constituting materials possessing dissimilar physicochemical (or even biological) properties, i.e., nanohybrid materials play a significant role. Nanoparticulate nanohybrids have gained a widespread reputation for prevention of viral crises, thanks to their promising antimicrobial properties as well as their potential to act as a carrier for vaccines. On the other hand, they can perform well as a photo-driven killer for viruses when they release reactive oxygen species (ROS) or photothermally damage the virus membrane. The nanofibers can also play a crucial protective role when integrated into face masks and personal protective equipment, particularly as hybridized with antiviral nanoparticles. In this draft, we review the antiviral nanohybrids that could potentially be applied to control, diagnose, and treat the consequences of COVID-19 pandemic. Considering the short age of this health problem, trivially the relevant technologies are not that many and are handful. Therefore, still progressing, older technologies with antiviral potential are also included and discussed. To conclude, nanohybrid nanomaterials with their high engineering potential and ability to inactivate pathogens including viruses will contribute decisively to the future of nanomedicine tackling the current and future pandemics.

## 1. Introduction

December 2019 was the onset of a new coronavirus pandemic in China that was then rapidly spread across the world. Two months later in February, the Coronavirus Study Group (CGS) of the International Committee on Virus Taxonomy (ICTV) designated the virus as SARS-CoV-2 and the caused disease was named as “coronoavirus disease 2019” (COVID-19) by the Director General of the World Health Organization (WHO) [1]. In March 2020, the WHO announced that the COVID-19 outbreak is a pandemic [1]. This rapidly spreading pandemic has affected all around the world, ending up with the infection of ~187 millions of people and the death of ~4.04 million of the infected cases (the statistics were obtained from worldometer.info on 10 July 2021).

Cough, fever, and fatigue are the main symptoms of the coronavirus disease and commonly seen in 68.7%, 85.6%, and 39.4% of the patients, respectively. The comorbidities including diabetes, hypertension, and coronary heart disease have been proved to directly affect the severity of the disease [2]. As reported lately [3,4,5,6], a number of patients suffering from COVID-19 shows damage not only in the respiratory system but also in kidney, heart, brain (encephalitis), and eye (conjunctivitis). Such adverse health consequences necessitate prompt development of vaccines, drugs, and medical tools that can effectively fight COVID-19. In this regard, nanomedicine and nanotechnology can offer many opportunities, as have done already throughout the history of novel medicine.

## 2. Biomedical Applications of Antiviral Nanomaterials

**Figure 1:**
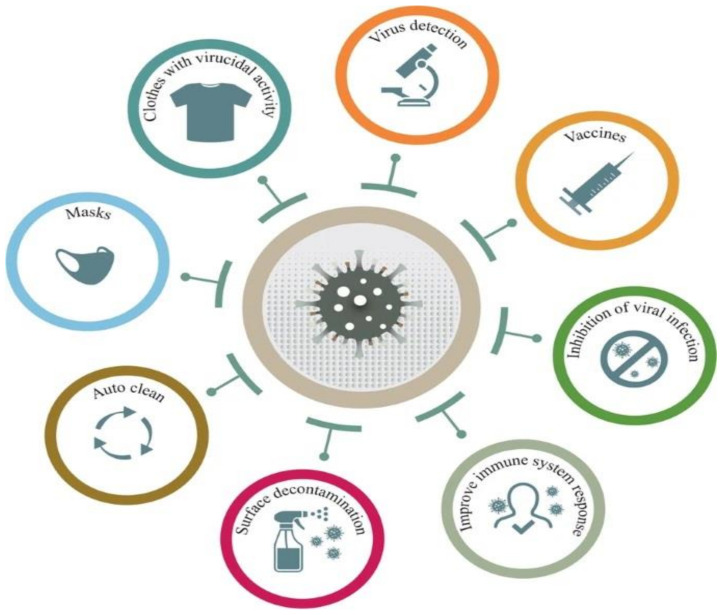
Biomedical applications of nanomaterials against the COVID-19 pandemic. Reproduced with permission [7] under a Creative Commons Attribution 4.0 International License.

Thanks to a variety of extraordinary electrical, chemical, magnetic, and antimicrobial properties as well as an extensive surface area/volume ratio, nanomaterials offer a diverse range of application in relevance to the COVID-19 pandemic. Figure 1 illustrates the biomedical applications of nanomaterials addressing different medicinal objectives such as control, prevention, diagnosis, and treatment.

Hybrid (composite) materials offer a favorable collection of properties originating from those of their distinct consituents, i.e., matrix and filler [8,9,10]. These properties could be beyond the predicted, classic properties and be indeed extraordinary, particularly, when dealing with nanofillers, as found in the nanohybrids. This field is dynamicaly growing and has been extensively highlighted worldwide.

**Figure 2:**
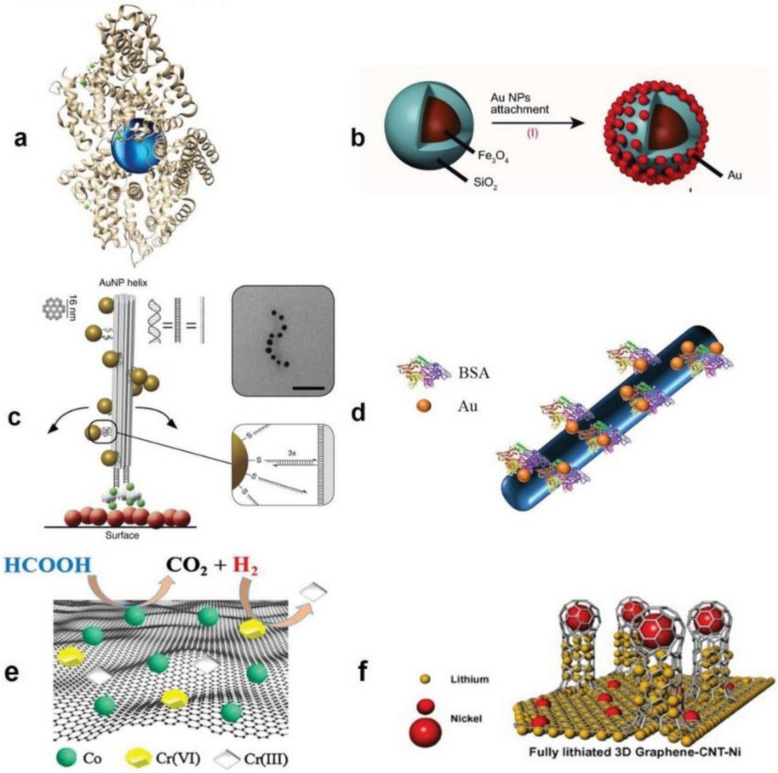
Nanohybrid materials in different dimensionalities, forms, and configurations. (**a**) A 0D bionanohybrid comprised of a plasmonic nanoparticle coupled with a protein, i.e., bovine serum albumin (BSA). (**b**) A 0D superparamagnetic core-shell nanohybrid composed of Au nanoparticles/Fe_3_O_4_@SiO_2_ developed via chemical immobilization of Au nanoparticles on the amino group functionalized SiO_2_ surface. (**c**) A 1D nanohybrid made of Au nanoparticle/DNA; in this system the Au nanoparticles (10 nm) are organized as a secondary left-handed helix located on the DNA origami bundle. The bundle *per se* consists of 24 parallel double helices. The magnified zone indicates that the thiolated ssDNA functionalized Au nanoparticles are coupled to the DNA origami. Next, functionalization of the DNA origami by the biotin groups (green) enables its attachment to a surface coated with BSA–biotin–neutravidin (red, green, and grey, respectively). Transmission electron microscopy (TEM) image exhibits a nanohelix mounted on a carbon-deposited grid (the scale bar represents 50 nm). (**d**) A 1D nanohybrid based on protein functionalized polymer nanofibers whereon Au nanoparticles are coupled by the protein ligands. (**e**) A 2D nanohybrid composed of cobalt-reduced graphene oxide (Co-rGO) (as shown in the figure, hexavalent chromium (Cr(VI)) is reduced by the nanohybrid). (**f**) A 3D hierarchical nanohybrid made of graphene–carbon nanotube–nickel (G-CNT-Ni), meant to operate as an anode material in the fully lithiated state. Reproduced with permission [8] Copyright 2018, Wiley-VCH.

Within the course of the past decade, thanks to recent technological advances, nanohybrids have been realized in new dimensionalities (0D–3D) and new compositions (especially involving the biological materials), Figure 2. Therefore, the definition of such advanced materials should be updated to include a more comprehensive view on the interplay of the constituting phases and their configuration. As a solid, ubiquitous definition, nanohybrids are multiphasic materials that show unique properties. These properties are tailored by the size (dimension), composition, and arrangement of the nanofillers. The latter could be notably influenced by the structure and characteristics of the encapsulating medium (material). In this study, we review biomedical applications of antiviral nanohybrids in relevance to COVID-19 pandemic and other viral crises in several classes of control, prevention, diagnosis, and treatment.

### 2.1. Nanohybrid Materials Used for Control/Prevention of Viral Transmission

**Figure 3:**
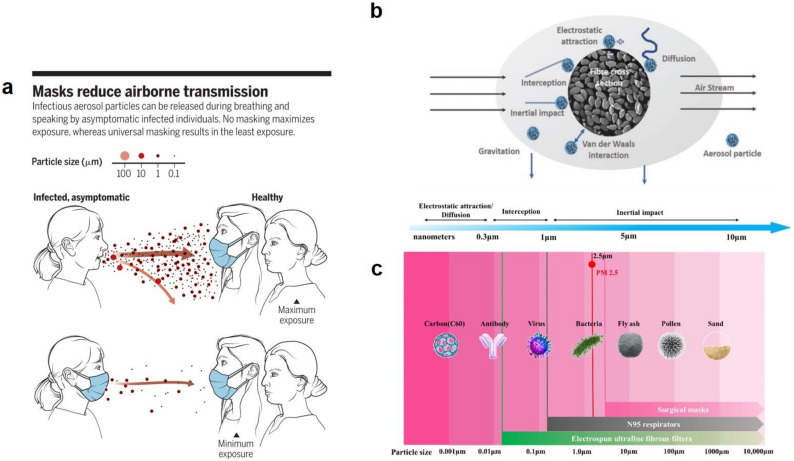
(**a**) airborne transmission of infectious aerosol particles is notably reduced by wearing masks. Reproduced with permission [17], Copyright 2020, Science. (**b**) Operation mechanisms of fibrous face masks depending on the size of the filtrate. (**c**) size (length) scale of different particles available in nature and the respective filtration means including surgical masks, N95 respirators, and electrospun fibrous filters. Reproduced with permission [18], Copyright 2021, Elsevier.

SARS-COV-2 is highly enduring and may attach on the surface of objects or persistently exist in the air as aerosols. According to various reports, coronavirus can survive and stay infectious for a sufficiently long period of time raising potential danger for spreading the virus from one place to another and especially for crowded places such as aircraft cabin, restaurants, and shopping centers [11]. In this regard, the main strategy to hamper the virus person-to-person transmission is wearing the protective equipment such as masks, gloves and protective clothing, Figure 3a. The conventional masks include the respirator masks (N95 and P2) and the surgical (face) masks. Respirator masks are particularly made to protect individuals against high-risk medical conditions in terms of presence of viruses and bacteria. Differently, surgical masks are meant to provide a lower level of such protection [12].

Nanohybrid materials can be implemented as the building blocks of the above mentioned personal protective equipment (PPE) to offer hydrophobicity and antimicrobial function, while maintaining breathability. The hydrophobicity of PPE can lead to formation of an effective barrier against deposition of the airborne droplets originating from cough or sneeze [11]. At the same time, Cu and Ag nanoparticles, for instance, can be applied on the surface of the PPE products to build up an antimicrobial barrier preventing the rapid growth of coronavirus on the surface. Moreover, the nanostructured, hybrid PPE materials, i.e., those implementing nanofibers alongside microfibers, can decrease the breathing resistance, thereby providing a comfortable wearing experience [11].

Recently, nanofiber based nanohybrids have been developed to improve the filtering performance of PPE against pathogens [13]. In this regard, Ag nanoparticles, CuO, iodine (I), titanium oxide (TiO_2_) with antimicrobial properties can confer the masks surfaces with a disinfection capacity for viruses [13]. The high surface to volume ratio of nanofibers can also enhance the capturing efficiency and provide some surface area- related phenomena including ion exchange and catalysis [14]. For instance, the nanofibers functionalized with chemicals and nucleating agents (e.g., o-iodosobenzoic acid (IBA) and β-cyclodextrin (β-CD)) can decompose or deactivate the contaminant, thereby minimizing the possibility of virus and pathogen inhale [15]. As a more sophisticated class of protective masks, filtering face piece respirator (FFPR) shows an improved performance in terms of air flow resistance and bacterial filtration efficiency. Such merits originate from co-presence of partially gelled sub-micron polypropylene (PP), PP nanofibers, and a hydrophilic bactericidal layer that can disinfect pathogens efficiently [16].

Apart from a high antimicrobial filtration efficiency, nanofibers can also optimize the wearing experience and offer a fitter mask choice through better facial seal. As a (nano)hybrid system, meltblown fibers and spunbond fibers can be combined with nanofibers through the electrospinning technology to form a basis for a particular type of respirators [18]. The nanofiber-based filtering facepiece respirators (FFR) can effectively capture viruses or pathogens via different mechanisms such as Brownian diffusion, because of their higher surface to volume ratio compared to that seen in the conventional masks. Moreover, the nanofiber FFR can be made thinner than N95 FFR, thus with higher air permeability and better breathability, leading to enhanced wearing experience [19]. The main filtration mechanisms of fiber-based face masks for three classes of particles in terms of size are shown in Figure 3b. The size range of the particles discriminated by electrospun fiber filters compared to conventional surgical masks and respirators is illustrated in Figure 3c. The face masks are designed to efficiently block macro, micro, and nanoparticles through interception, inertial impaction, and diffusion, respectively [12]. Particularly, inertial impaction matters most, given the size range of viruses and bacteria.

The particles smaller than 300 nm, hardly collide with the pore walls and are readily bombarded by the surrounding air molecules. To capture these particles, electrospun nanohybrid nanofibers have been proved to be very efficient. Xianhua et al. [20] prepared a nanohybrid nanofiber mask based on Ag nanoparticles reinforced polyvinyl alcohol (PVA) nanofibers through electrospinning. The as-formed nanohybrid nanofibers were deposited on an activated carbon nonwoven fabric as the substrate. As verified in this study, the nanohybrid protective mask is able to show an excellent performance in terms of filterability, moisture permeability, and breathability. Such characteristics depend mainly on the Ag nanofiller amount, so that with the increase of filling factor, the nanohybrid nanofiber layer turns thicker and thus lowers the air permeability. On the other hand, under same conditions, the moisture permeability increases. Zhang et al. [21] devised an antiviral nanofibrous membrane composed of vitamin k incorporated poly(vinyl alcohol-co-ethylene) that could show intensive photoactivity, thereby generating ROS under solar and UVA light. As a result of such a performance, the nanofiber membrane was able to inactivate >99.9% of viruses and thus offer a promising capacity for development of PPE and face masks.

Apart from the use of PPEs, vaccination has ever been considered as a successful prevention strategy. In this regard, nanomaterials as efficient carriers can transport the vaccine into proper cellular colonies and subcellular areas. Given the size and dimensionality, viruses are in fact living nanomaterials and the Live Attenuated Vaccines (LAVs), Inactivated Vaccines (IVs) and viral vectors inspired from viruses are nanotechnologies. Performing in the same size scale as viruses, nanoparticles can play a crucial role in the development of vaccines and thus immunoengineering [22]. Man-made and natural nanoparticles resemble viruses in terms of structure, while biotechnology, nanochemistry, and chemical biology synergistically enable creation of advanced vaccines that will be incorporated therein.

**DNA vaccine carriers**: DNA vaccine’s working mechanism is based on inclusion of a protein antigen’s gene in a recombinant eukaryotic expression vector (e.g., a plasmid) and its subsequent introduction into the body to generate the exogenous antigen, driving antigen-specific immune reactions against the disease [23]. Versus traditional vaccines, these vaccines imitate natural infections and provoke antibody secretion as well as the cell mediated immune reactions. Therefore, they hold promise for treatment of chronic viral infection and cancer. Furthermore, the DNA vaccine assures reliable gene expression in transfected host cells, thus producing enough quantity of antigen. Facile manipulation of genetic sequences through recombinant methods, large scale production and simple storage and transport (no need to cold-chain), the potential of induction of particular antigen and immunoregulatory proteins, and most importantly proven safety in animal/human clinical tests are of other advantages of the DNA vaccines [24,25,26]. However, such vaccines show insufficient immunogenicity and due to enzymatic (nuclease) degradation and subsequent elimination through the reticular endothelial system, their in vivo half-life is quite short (minutes) [27]. Additionally, naked DNA must be able to defeat several cellular and subcellular hurdles such as the plasma membrane to induce protein expression [28]. Therefore, efficient transportation of DNA vaccine specifically to the desired cells in different organs and lymphoid tissues and assuring reliable, optimum gene transfection efficiency along with induction of the maturation of the dendritic cells and presentation of antigen are the main objectives towards the creation of DNA vaccines that could be properly met by introduction of new, advanced biomaterial based carriers [29].

Different kinds of man-made nanocarriers such as polymeric nanoparticles and cationic liposomes have been synthesized for the DNA vaccine delivery through cell membranes [30]. For instance, Zhao et al. [31] designed a nanocarrier composed of poly (lactic-co-glycolic) acid (PLGA) coated with chitosan (CS) to deliver the DNA vaccine against Newcastle disease virus (NDV). As stated by the authors, the chitosan coated PLGA nanocarrier of the DNA vaccine assures a higher cellular, humoral, and mucosal immune response in a safer and efficient manner. Farris et al. [32] encapsulated DNA vaccine/chitosan nanoparticle within a zein (ZN) microparticle and in fact devised a hybrid-dual particulate carrier to govern the DNA vaccine delivery. Routinely, the DNA vaccines that are delivered orally target the mucosa (i.e., intestinal mucosa) to cause the mucosal immunity. However, when the DNA vaccines pass through the gastrointestinal tract, it would be readily disintegrated at a low pH, and by endogenous nucleases and gastric enzymes. The designed hybrid-dual particulate carrier shows a more controlled DNA release behavior in simulated gastric fluid (SGF) as compared to DNA/CS nanoparticles. Additionally, the external ZN matrix degrades at specific areas and thereby leads to successful mediated transfection of the DNA/CS nanoparticles *in vitro*. Eventually, the hybrid-dual particulate carrier encoding GFP could drive the production of anti-GFP IgA antibodies, verifying in vivo transfection and expression. Layek et al. [33] developed a DNA vaccine nanocarrier made of mannosylated phenylalanine grafted chitosan (Man-CS-Phe) as a possible immunotherapeutic route for chronic hepatitis B. Within such cationic micelles, plasmid DNA is condensed as nanosized polyplexes. As a result, the complexed DNA is shielded against enzymatic (nuclease) degradation.

**Figure 4:**
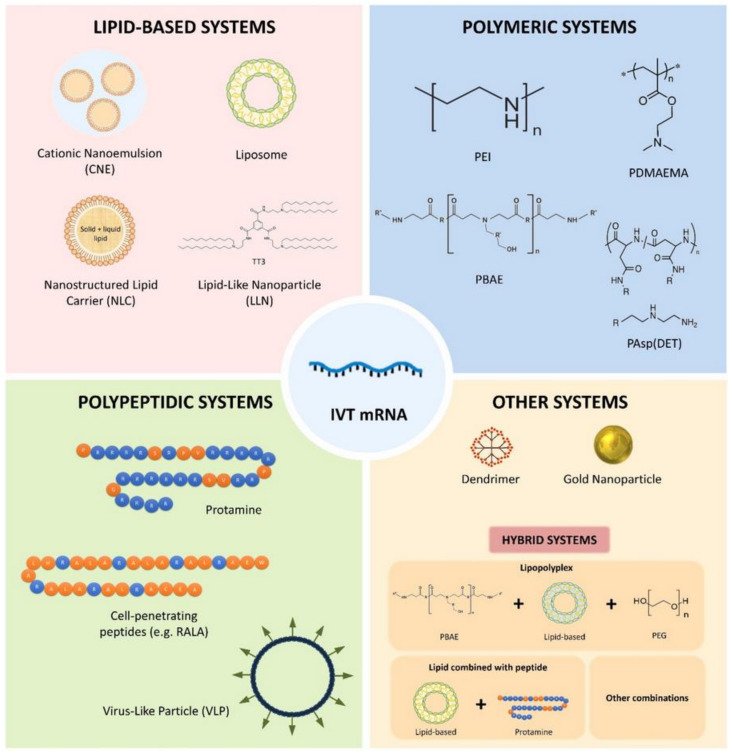
The as-yet studied mRNA vaccine nanocarriers. Reproduced with permission [38].

**mRNA vaccine carriers**: the mRNA vaccines have also found notable application with respect to prevention of viral infections. As currently seen in the case of the COVID-19 mRNA vaccines, e.g., the one produced by Moderna, lipid nanoparticles are crucial in effective protection against enzymatic (ribonuclease) degradation and transportation of mRNA to cells [34]. Other than lipid nanoparticles, several new nanotechnologies have been also devised for the sake of delivery of mRNA vaccines, Figure 4. Nanomaterial based technologies such as dendrimers, cationic nanoemulsions, polysaccharide particles, or liposomes have been also created to raise the stability of mRNA vaccines and optimize their delivery mode [35].

Elia et al. [36] designed an mRNA vaccine holding lipid nanoparticle system. In this mRNA nanocarrier, the lipid nanoparticles enclosed SARS-CoV-2 human Fc-conjugated receptor-binding domain (RBD-hFc). After intramuscular administration of the mRNA nanocarrier, a notable humoral reaction, a Th1-biased cellular behavior in BALB/c mice, as well as a large extent of neutralizing antibodies were recorded. This strategy, i.e., encapsulating mRNA vaccine into lipid nanoparticles has been also applied in relation to other types of viruses. For instance, Zhuang et al. [37] incorporated mRNA vaccine (coupled with in vitro transcription (IVT)) into cationic lipid nanoparticle as an H1N1 influenza virus vaccine nanocarrier and compared its gene delivery efficiency with that of mannose-conjugated lipid nanoparticle. As they reported, the latter nanocarrier was more successful in this respect than its counterpart both in vivo and *in vitro*. The most optimum system, IVT-mRNA-n3 loaded mannose-lipid nanoparticle was proved to be efficiently applicable for immunization of C57BL/6 mice against H1N1 influenza virus.

**Subunit vaccine carriers**: Subunit vaccines comprise very limited structural components of SARS-CoV-2, able to induce protective immune reactions in the body, upon administration alongside molecular supports, thereby raising immunogenicity [22]. The subunit vaccines can be made via incorporation of viral proteins in protein cages, virus-like particles (VLPs), and synthetic nanomaterials, performing as delivery carriers and/or adjuvants [39,40,41]. For instance, influenza protein haemagglutinin incorporated liposome is the basis of the influenza virus vaccine Crucell (Janssen, Johnson & Johnson) [42]. The subunit vaccine nanocarriers provide antigen multivalency and allow optimum concurrent delivery of adjuvant and antigen to secondary lymphoid organs [43]. Furthermore, thanks to their specific size scale, they enable lymphatic trafficking and favorable uptake by the antigen presenting cells (APCs). They induce depot effects for stable immune stimulus and ease the antigen cross presentation, that allows the extracellular antigens to be presented via the MHC-I pathway for CD8^+^ T cell engagement [44].

### 2.2. Nanohybrid Materials Used for Biomedical Waste Management

As the pandemic spreads all over the world, a large number of PPE is being consumed every day. Therefore, management of biomedical wastes is crucial to avoid an extra pressure on the environment and spread of the virus [45]. The biomedical waste differs notably from the normal waste considering the likely presence of pathogens on its surface that could lead to further contamination and release of viruses and bacteria [45].

The contaminated healthcare wastes are typically discarded in landfills, thus potentially might engender pollution of surface, drinking and ground water resources, in case the landfill has been improperly designed. The healthcare wastes can be chemically treated by chemical disinfectants, thereby further contaminating the environment via release of hazardous chemicals into the environment. As another option, waste incineration has been largely taken into practice. Yet, incomplete incineration could potentially pollute the air and produce ash remainder. The other conventional methods in management of biomedical waste include mechanical methods involving granulation, crushing, pulverization, grinding, shredding, agitation, and mixing. These approaches do not necessarily eliminate the pathogen related contaminations, but lower the waste mass thus ease its subsequent processing or disposal. Chemical disinfection, e.g., via implementation of chlorine compounds, is another popular approach to inactivate the pathogens in medical waste, and also to oxidize harmful chemical materials. Microwave radiation and its resulting generated heat can also potentially be utilized to treat medical waste. Thanks to detrimental effect of gamma rays on pathogen’s DNA, gamma irradiation caused by radioactive isotopes of cobalt, shows a high potential for sterilization of waste. However, the shadowing effect is challenging and the waste surfaces directly exposed to the radiation source become more sterile than those located on the shaded side [45].

**Figure 5:**
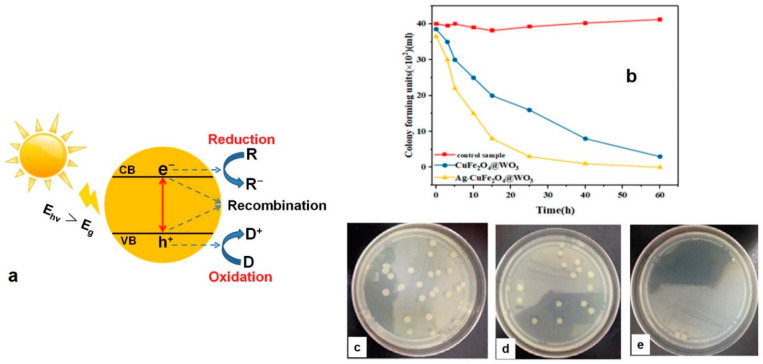
(**a**) Schematic illustrates the solar light driven photocatalysis (*E_hv_* and *E_g_* are the solar light photon energy and band gap energy, respectively, and, CB and VB are the conduction and valence band, respectively. R and D are the electron acceptor and electron donor, respectively. Reproduced with permission [68], Copyright 2017, Elsevier. (**b**) The Ag-CuFe_2_O_4_@WO_3_ nanohybrid nanoparticles kill *E. coli* within the course of a 60 h incubation period upon exposure to UV light. The density of the bacteria colonies imaged in the absence of the nanohybrid nanoparticles (**c**), and in the presence of CuFe_2_O_4_@WO_3_ (**d**) and Ag-CuFe_2_O_4_@WO_3_ nanoparticles (**e**). Reproduced with permission [67].

Advantageous over the mentioned management techniques in terms of cost-efficiency, sunlight based photocatalysis has been developed for both solid and liquid wastes [46]. This approach is drawing more attention for environmental remediation, considering the demand to obtain the highest degradation efficiency of contaminants possible under affordable conditions in terms of temperature and pressure. The main driving force in this treatment is the near-UV light (whose wavelength varies from 400 nm down to 300 nm) that could be even replaced by sunlight to render the technique more economical. As shown in Figure 5a, sunlight driven photocatalysis in the presence of a nanophotocatalyst enables release of hydroxyl radicals (OH•). Having unpaired electrons, OH• notably oxidizes the resistant organics (e.g., pathogens) [47]. Owing to the abundance of low cost, efficient photocatalysts, photocatalysis is of the most renowned dissociation process for organic pollutants. This type of pollutants is easily decomposed to water and CO_2_ in a liquid medium under the influence of the photocatalytic activity of a semiconductor metal oxide nanoparticle, e.g., TiO_2_ [48].

Despite several advantages of the photocatalysis process for waste management including low energy consumption, ecofriendliness with insignificant chemical input, production of no harmful byproducts (in general least amount of secondary waste is produced), mild operational conditions (temperature and pressure), and versatility in terms of operating medium (solid, liquid, and gas), the applicability of the process is limited due to charge separation, interfacial charge transfer and charge carrier recombination [10,49,50,51]. One promising solution for such challenges and also extending applicability of the nanophotocatalysts is their hybridization with other supplementary, supportive components. For instance, Cu-deposited TiO_2_ photocatalysts have been proposed to offer biocidal activity and cooperative effect of photocatalysis and lethality of copper [52]. Such a nanohybrid system was able to deactivate *E. coli* bacteria when irradiated with very weak UV light. It has been claimed that the photocatalysis process leads to destruction of the external membrane in the cell envelope, thereby allowing the Cu ions to get into the cytoplasmic membrane. The copper ions damage the cytoplasmic membrane and engender the cell’s disintegration.

In general, photocatalysis can provoke degradation of simple compounds (proteins and DNA), thereby imposing an inhibitory effect on viruses and bacteria [53]. The photocatalytic detrimental impact takes place at two levels [54]: (1) Photo-inactivation ending up with a disinfectant effect, and (2) Decomposition of viral cells bringing about a sterilizing effect. Despite validation of effectiveness of the photocatalytic systems for virus inactivation via various laboratory experiments involving several types of microorganisms, the antiviral mechanism is still to be understood [55,56,57]. The whole process seems to initiate with adsorption of the virus on the photocatalyst surface, followed by an oxidative radical attack on the capsid protein and on virus binding sites, aka, direct attack—redox type. As explained in [58], the inactivation process of viruses is governed by the presence of radicals and particularly reactive oxygen species (ROS) such as °OH, •O_2_^−^, HO_2_•, and H_2_O_2_ that are available in the bulk phase and are independent of the catalyst. As similarly mentioned for the *E. coli* bacteria, the decomposition process continues with destruction of the cell wall and the cytoplasmic membrane, induced by the generation of ROS that can result in the release of cellular content, cell lysis and eventually total mineralization of the organism [54].

Given the same inactivation mechanism of viruses and bacteria, the photocatalytic nanohybrids with effective antibacterial performance can potentially be applied for disinfection (virus removal) of biomedical wastes. For instance, CdO-MgO [59], CeO_2_/CdO [60], PbS-CdO [61], CdO-NiO [62], CdO-ZnO [63], NiO-CdO [64], ZnO/MgO [65], CuO-MgO [66] nanohybrids are some examples for the photocatalysts able to show antimicrobial performance. In this regard, Sayadi et al. [67] devised a photocatalytic nanohybrid composed of tungsten trioxide (WO_3_) hybridized with Ag doped CuFe_2_O_4_ that could offer a photocatalytic activity when exposed to UV/visible light and be recovered from water efficiently thanks to the excellent photocatalytic and magnetic properties of the mentioned components, respectively. Due to co-existence of Ag, this nanohybrid photocatalyst was able to inactivate the *E. coli* bacteria when UV irradiated and after 12 h incubation, Figure 5b–e. At the interface of the WO_3_ and CuFe_2_O_4_ phases (core-shell structure), the electrons supplied by Ag are captured by the O_2_ molecules, and consequently generate reactive species including OOH◦, O_2_, and HOH that optimally inactivate the bacteria.

### 2.3. Nanohybrid Materials Used for Early Diagnosis of Virus Infection

To accurately diagnose the presence of viruses, advanced, reliable technologies able to detect viruses with outstanding sensitivity are now needed more than ever. The conventional PCR-based diagnosis technology, that is routinely employed for virus detection necessitates operators and are costly in terms of equipment, challenging its implementation in the areas poor in resources [69,70]. Additionally, the technologies relying on antigen–antibody reactions, such as immunochromatography, are not sufficiently sensitive [71]. Therefore, it is indeed questionable to target accurate detection of viruses to prevent their spread based on the techniques that are susceptible to false negatives and false positives [72].

Thanks to the expansive specific surface area of nanomaterials, the detection technologies employing nanomaterials can potentially achieve precise analyte-specific detection signals with superior sensitivity to even trace amounts of viruses [73,74]. In this regard, virus detection can be optically carried out by provoking a fluorescence-raising effect on virus when the adjacent plasmonic nanoparticles create a localized surface plasmon resonance (LSPR) effect [75]. The as-emerging optical properties of the nanoparticles highly depend on their interparticle spacing [76]. In addition to plasmonic nanoparticles, superconductive nanomaterials can be also employed in development of highly sensitive electrochemical biosensors for virus detection [77,78].

The Dengue virus has been similarly detected using nanohybrid optical sensors. In this regard, the optical sensors composed of graphene oxide (GO) have drawn interest because of their fascinating characteristics such as high density of functional groups, thinness, negligible mass, extensive specific area, a structure providing high π-conjugation, and optimum robustness [79,80,81]. The presence of a large number of functional groups on GO, however, could be challenging with respect to its electron transfer ability, given that the functional groups can potentially disrupt conductive zones and even the largely oxidized GO is extremely poor in terms of electrical conductivity [82]. To resolve this issue, yet maintaining beneficial properties, GO is typically reduced and its oxidized functional groups are discarded, ending up with formation of reduced graphene oxide (rGO), that is an electrically conductive material, can be stored for a longer period without agglomeration, and is more chemical resistant (endures in the organic solvents) [83,84]. Additionally, the residual oxygen bearing functional groups of rGO can be employed for the purpose of chemical functionalization and creation of nanohybrid materials. As an example, rGO can be further functionalized using primary amines (-NH_2_) to get hydrophilized and to allow adhesion and binding of analytes, e.g., viruses [85,86]. In this relevance, globular dendrimer of polyamidoamine (PAMAM) has been coupled with rGO to realize a highly sensitive detection platform. PAMAM dendrimers are notably useful for various sensing purposes, thanks to their efficient transporting behavior for bioactive agents and their non-toxicity [87,88]. Omar et al. [89] devised an optical sensor, performing based on the SPR effect, for detection of the dengue virus. The nanohybrid sensor was composed of monoclonal antibody decorated dithiobis (succinimidyl undecanoate, DSU)/amine-functionalized rGO–PAMAM. The as-developed sensor was able to accurately record the alterations of the SPR angle, for instance, for the virus concentrations as low as 0.08 pM in 8 min. Interestingly, the sensor behaved selectively, and the sensitivity to other types of proteins was lower than that to the virus. Highly conductive substrates made of graphene have been hybridized with Au to form a sensing platform (overlaying a Au screen printed electrode) for electrochemical detection of influenza virus. The graphene-Au nanohybrid biosensor performs based on quantification of neuraminidase (N) activity [90].

**Figure 6:**
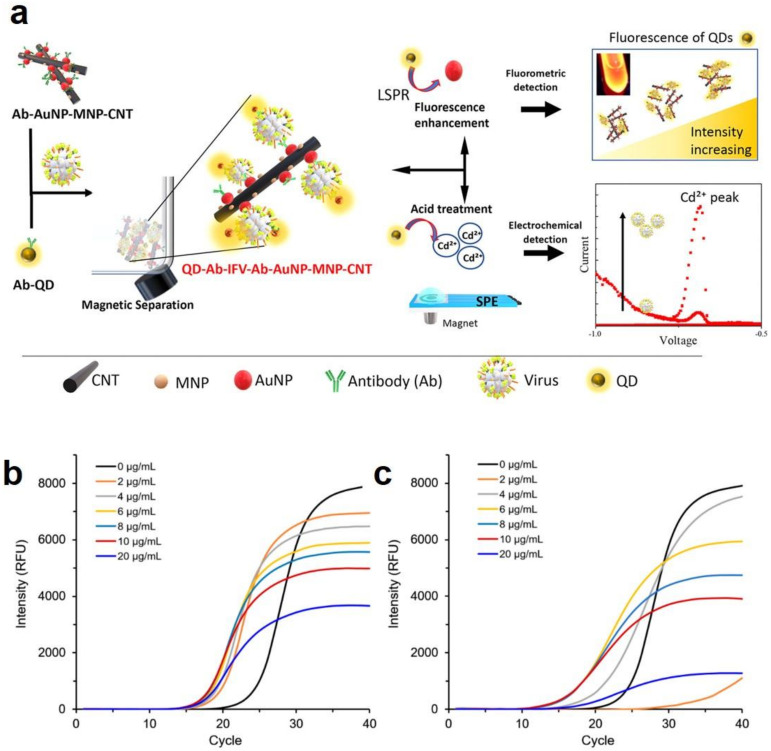
(**a**) Schematic illustration of the optical/electrochemical detection process of the Influenza virus based on implementation of plasmonic nanocomposites and quantum dots. Reproduced with permission [94], Copyright 2021, American Chemical Society. The improvement of the PCR efficiency (nano-PCR based on pan-type primers) through inclusion of GO-AuNPs at different concentrations for the purpose of quantification of FMDV O-type (**b**) and FMDV A-type (**c**). Reproduced with permission [95].

Despite the mentioned merits, nanomaterial-based virus detectors suffer from lack of stability and reliability of the detection signal [91]. Additionally, high sensitivity imposes a high concern of producing nonspecific signals. To prevent generation of nonspecific signals, thereby enabling a more reliable and stable detection process, two different signals can be based [92]. Therefore, highly sensitive and reliable nanomaterial-based biosensors should be developed that generate two signals. To achieve such a feature and develop a dual-signal virus detector, the nanomaterials able to efficiently generate and enhance electrochemical and optical signals, such as gold nanoparticles (AuNPs) are of paramount importance [93]. In this relevance, Takemura et al. [94] have designed a novel technique for detection of the Influenza virus based on the electrochemical and optical signals. The detection performance tightly depends on the virus concentration. The virus detector is in fact a nanohybrid system that comprises of: (1) plasmonic (Au) nanoparticles (AuNP) to reinforce the fluorescence signal of CdSeTeS quantum dots (QDs) through the LSPR effect (noteworthy, the virus could bound onto the plasmonic nanoparticles via antibody (Ab)), (2) magnetic nanoparticles (MNP) to separate the analyte stuck within the nanohybrid structure, and (3) carbon nanotubes (CNT), meant to act as a matrix to integrate all the components coupled with the antibodies of the Influenza virus. The mentioned QDs suspending in the solution were responsible for generation of the optical signal, particularly in close proximity of the Au nanoparticles, thanks to their long fluorescence lifetime, and were able to release Cd ions at low pHs and thereby produce an electrochemical signal, Figure 6a. In more precise words, when the QD–Ab/virus/Ab–AuNP–MNP–CNT nanoassembly is dissolved at a low pH, the Cd ions are released from the QDs, whose concentration is quantified electrochemically by the AuNP–MNP–CNT-deposited carbon electrode.

As mentioned earlier, despite popularity of PCR as a detection technique for viruses, it suffers from poor specificity, insufficient sensitivity, and generation of false positive results. To address such shortcomings, metal and carbon nanomaterials are included to raise the quality of performance of PCR assays. Kim et al. [95] enhanced the capability of PCR using a nanocomposite made of GO sheets (as the matrix) and AuNPs (~15 nm) to enable sensitive diagnosis of the foot-and-mouth disease virus (FMDV), which leads to a drastically infectious and fatal viral disease for the animals with cloven-hoof such as pigs, and thus negatively impacts on the swine industry. As validated by the authors, the limit of detection (LoD) of real-time PCR upgraded by GO-AuNPs raised up to ~1000 times. The improvement in the PCR detection efficiency was determined via comparison of DNA amplification potential in the presence (at various concentrations) and absence of GO-AuNPs. As shown in Figure 6b, the optimal concentration was proved to be ~10 µg/mL, particularly, 20 µg/mL of GO-AuNPs led to a notable decline in signals. It is known that GO-AuNPs can electrostatically attach to single-stranded DNA (ssDNA) in a selective manner [96]. As a result, the quality of PCR performance is notably enhanced when acting as a single-stranded DNA binding protein (SSB), optimizing the interaction between templates and primers while DNA replicates *in vivo*. On the other hand, larger GO-AuNPs concentrations could potentially impede the PCR reaction, due to their tendency to bind to double-stranded DNA (dsDNA), rather than to ssDNA.

### 2.4. Nanohybrid Materials Used for Viral Infection Therapy

Nanohybrids benefitting from a therapeutic agent coupled with a supportive organic/inorganic nanomaterial have shown promising applicability for treatment of various viral infections caused by Influenza, Hepatitis C, and SARS-CoV-2.

Influenza A virus (IAV) is accounted as an important infectious pathogen in relation to humans and animals and frequently engenders epidemics and epizootics. Annually, influenza infects around 100–500 million people, thereof almost 500,000 die [97,98]. The exclusive structure of this virus, with respect to genome fragmentation (the genome comprises eight negatively charged segments of single-stranded RNA) and glycoproteins diversity, is the main ground for its global spread. As a consequence of gene recombination and antigenic shift, new variants of the Influenza virus emerge and bring about pandemics and epidemics [99]. To address the global challenge caused by the spread of Influenza, only a limited variety of drugs with selective performance such as neuraminidase inhibitors (e.g., oseltamivir) and adamantane drugs are available that could even drive the formation of resistant IAV strains and side effects [100]. Therefore, there is a demand for development of new therapeutics that can deactivate the influenza virus in an effective manner. In this regard, the nucleic acid fragments that can identify the target nucleic acids selectively, thereby minimizing adverse side effects in comparison to non-specific traditional drugs, are promising candidates. Despite such merits, these therapeutics have not found extensive application mainly due to their poor serum stability and their challenging diffusion into cells. To address the former bottleneck, i.e., stabilization of oligonucleotides, chemical treatment has been proved to be efficient [101]. On the other hand, liposomes, virus vectors, cationic polymers, transporting peptides, among others can act as carriers to ease and improve delivery of oligonucleotides [102,103,104]. However, even the most successful delivery approach is not efficient enough and might impose toxicity. To advance this research area and to meet the need to effective delivery systems, Levina et al. [99] proposed a new platform able to deliver nucleic acid fragments (ODN) into eukaryotic cells based on a nanocomposite material. The nanocomposite comprised of ODN loaded polylysine that was non-covalently immobilized onto TiO_2_ nanoparticles. The selected nanoparticles are biocompatible and non-toxic and enable the ODN delivery via the cell membrane [105,106]. Moreover, they stabilize and protect the ODN against intracellular enzymes [107]. Upon entry into cells, the carried oligonucleotides are released from the nanocomposites in the cytoplasm or penetrate into the nuclei and bind to the RNA molecules [108]. As verified via in vitro tests, the nanocomposites induce no toxicity reaction, readily enter into the eukaryotic cells, and significantly deactivate 3 IAV subtypes, e.g., hazardous H5N1 avian influenza.

Hepatitis C virus (HCV) is another important virus affecting more than 58 million people worldwide and resulting in severe liver disease [109]. Thanks to further comprehension of the biology of HCV as well as identification of antiviral targeting of vital functions of the virus, the relevant treatments are being advanced and becoming more efficient [110]. In this regard, the NS3 and NS5A proteases and NS5B polymerase are regarded as important targets for the creation of direct-acting antiviral drugs [111]. The standard therapy for the HCV genotype 4a comprises ledipasvir, ombitasvir, and sofosbuvir with a response rate of over 90% [112]. Although this treatment has shown considerable promises, its efficacy is below optimum and undesired consequences such as photosensitivity, pruritus, rash, anemia, etc. [113] could potentially emerge. Additionally, the rising number of patients who suffer from dangerous liver diseases with no proper treatment choice further highlights the importance of development of suitable therapeutic approaches. Challenging fulfilment of this objective, viral RNA is rapidly mutated and thereby creation of state-of-the art anti-HCV drugs are hampered. One promising solution for treatment of the HCV infections is indeed the use of plant derived natural compounds that show relevant antiviral performance [114]. Furthermore, such compounds exhibit hepatoprotective effect due to their natural substances including caffeine, naringenin, silymarin, and epigallocatechin-3-gallate (EGCG) [115,116]. As an example, for the plant-derived antiviral compound, turmeric curcumin, aka, *curcuma longa*, has shown promising therapeutic efficiency with no particular side effects [117]. Curcumin offers antiviral effect against hepatitis B virus [118], influenza, human herpes, and HIV viruses [119]. As reported by Pecheur [120], curcumin can also deactivate HCV through challenging the viral adhesion and fusing to hepatocytes and inhibiting intercellular transmission by damaging the membrane’s structure. Nevertheless, curcumin is poorly soluble and hardly penetrated into cells. One solution to overcome such problems, is the use of polymeric nanoparticles as a carrier for curcumin, that assures its steady release to infected cells and its bioavailability, and hampers its degradation, thereby enhancing its therapeutic capacity [121]. In this regard, biocompatible polymers originating from nature are highly demanded. For instance, chitosan, a derivative of chitin, is a natural biopolymer (polysaccharide) and after cellulose is ranked the second most available polysaccharide in nature [122]. Chitosan shows immunogenicity, and anticancer, antimicrobial, and anti-inflammatory effects [123]. Chitosan nanoparticles are non-toxic at low dosages (30 µg/mL) and offer an anticancer effect at high dosages (>100 µg/mL). These promising potentials encourage its implementation as a drug carrier [124,125]. In this regard, Ramana et al. [126] employed chitosan nanoparticles as a carrier for antiretroviral agents such as saquinavir, that is a protease inhibitor, to deactivate HIV. By this strategy, cell targeting efficiency raised up to 92% in comparison with that of the soluble drug alone. In another study, Loutfy et al. [127] studied the antiviral efficiency of curcumin-chitosan nanocomposite against HCV-4 *in silico*, involving the hepatoblastoma cells. According to their results, the nanocomposite system is able to notably reduce the HCV core protein expression, as verified by the Western blot assay. Such reduction was much superior in the case of the nanocomposite as compared to the controls including curcumin and chitosan nanoparticles, implying its extraordinary antiviral effect via blocking the penetration or replication of virus. Though, the achieved results clearly demonstrate the applicability of the nanocomposite against viral infection, the anti-HCV effect of the nanocomposite in the replicating system and its therapeutic index need to be evaluated. Moreover, the nanocomposite carrier should be also precisely tested *in vivo*.

**Figure 7:**
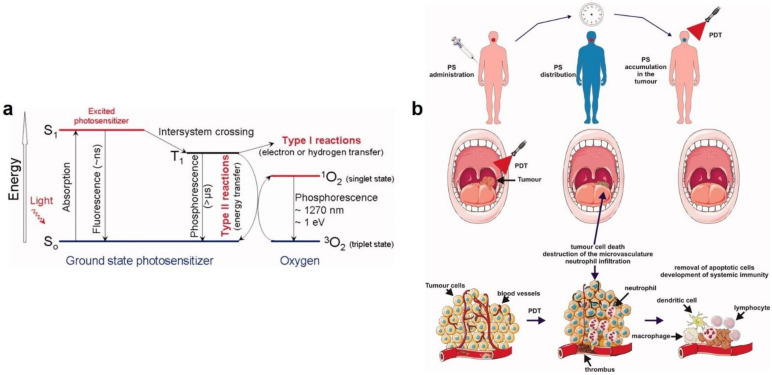
(**a**) The mechanism of photosensitization as described through a Modified Jablonski Diagram. Upon light irradiation, a photosensitizer (PS) molecule undergoes a transition from the ground singlet state (S_0_) to a provoked singlet state (S_1_). At S_1_ the molecule might experience intersystem crossing towards an excited triplet state (T_1_). Subsequently, it either creates radicals (Type I process) or pass its energy on a molecular oxygen (^3^O_2_), forming singlet oxygen (^1^O_2_), i.e., the main cytotoxic substance taking part in PDT. In the diagram, ns, μs, nm, and eV represent nanoseconds, microseconds, nanometers, and electron volts, respectively. (**b**) The schematic illustration of PDT for cancer therapy, where a PS is distributed topically or systemically within body. Over time, PS is selectively accumulated in the tumor and then by irradiation is excited. Subjected to molecular oxygen, a photochemical process is carried out that leads to formation of singlet oxygen (^1^O_2_). Severe destruction of cellular macromolecules causes the cell death of tumor through a necrotic, autophagic, or apoptotic process, that is complemented with inflammation. An acute local inflammatory process eliminates the dead cells and restores normal tissue homeostasis. Reproduced with permission [130], Copyright 2011, American Cancer Society.

In addition to the aforementioned drug-based antiviral therapies, photodynamic therapy (PDT) is an efficient strategy to deactivate viruses including SARS CoV-2. In this approach, the target cells are attacked when photosensitive agents, aka, photosensitizers (PSs), are excited by light irradiation and thereby produce reactive oxygen species (ROS) that are fatal to cells [1]. In fact, ROS seriously damages the nucleic acids and proteins of virus [128]. Figure 7a shows the mechanism of generation of ROS by PSs. Majority of PSs in their ground (i.e., singlet) state possess 2 electrons, spinning oppositely in a molecular orbit with the most optimum energetic level. Upon light absorption, one of these electrons jump to an orbit with a higher energy state. As a result, the PS becomes extremely unstable and starts to emit the extra energy in the form of heat and/or fluorescence. As a second scenario, the PS after excitation might experience an intersystem crossing, thereby forming a triplet state with higher stability where one electron inversely spins. To revert to the ground state, the PS should either lose energy without emitting radiation or passing the extra energy on molecular oxygen (O_2_), ending up with generation of ^1^O_2_ (Type II process) [129]. PS can also react with an organic substance within a cellular medium, whereby it receives an electron or a hydrogen atom and converts to a radical (Type I process). The as-reduced PS is subsequently autoxidized and releases a superoxide anion radical ($O_{2}^{•-}$). The generated radical undergoes one-electron reduction thereby forming hydrogen peroxide (H_2_O_2_), that is subjected to one-electron reduction, leading to creation of a highly oxidative hydroxyl radical (HO^•^). It is thought that majority of PSs perform through Type II process to generate ROS [130].

PDT was for the first time in 1970s used for the purpose of deactivation of viruses [131]. Beforehand, it was mainly employed for clinical treatment of different oncological disorders, Figure 7b [130]. With respect to virotherapy, PDT has shown applicability against herpes simplex virus, human immunodeficiency virus, and human papilloma virus [128,131,132]. However, some challenges regarding the PSs including their typical hydrophobicity, leading to their aggregation in water-based solutions, lack of target specificity, and also insufficient penetration into the desired tissue have notably restricted clinical application of PDT [1]. To resolve such issues, Lim et al. [133] developed sodium yttrium fluoride (NaYF_4_) upconversion nanoparticles surface decorated with zinc phthalocyanine PSs. To hydrophilize the nanohybrid, the nanoparticles were coated with polyethylenimine (PEI). Such a nanohybrid was able to deactivate adenovirus type 5 and Dengue virus serotype 2, representative of nonenveloped and enveloped viruses, respectively. Curcumin loaded liposome is another nanohybrid system developed to inactivate viruses (papillomavirus) through release of curcumin alongside PDT [134]. In this system, liposomal encapsulation enables optimal, targeted delivery of curcumin to cells. On the other hand, curcumin liposomes were able to induce a PDT (phototoxic) effect thus cell death in the papilloma virus-related tumor cell lines.

Another relevant approach is photobiomodulation therapy (PBMT), i.e., the light therapy based on non-ionizing types of light sources such as laser, that could be employed with or without application of a static magnetic field (sMF) [135,136]. Severe cases of COVID-19 arise when immune response in the patient’s body is insufficient and pro-inflammatory cytokines are extensively released and harm different organs such as lungs. As a supportive therapeutic strategy, PBMT utilizing a low-level laser (LLLT) raises immunity, stimulate tissue regeneration and decline pro-inflammatory cytokines [135]. De Marchi et al. [137] studied the treatment of the patients suffering from severe COVID-19, who were in the need of mechanical ventilation, through PBMT-sMF. As they reported, PBMT-sMF could enhance the ventilatory parameters and improve the immune response. Conclusively, PBMT-sMF or LLLT may show a proper therapeutic effect, thereby reducing the medical burden imposed on the healthcare systems and alleviate the application of inadequate medicinal resources during the COVID-19 pandemic.

### 2.5. Nanohybrid Materials Used as Antiviral Coatings

In order to decline the chance of spread of viruses, we need to cut off their transmission routes by applying antimicrobial nanomaterials on the surfaces and creating antiviral coatings. In this regard, different antiviral metal ions including Au, Ag, and Cu, and metal oxides, antiviral polymers, and new antiviral nanomaterials have been studied to be used as virus inactivating coatings [138,139,140,141,142,143].

**Figure 8:**
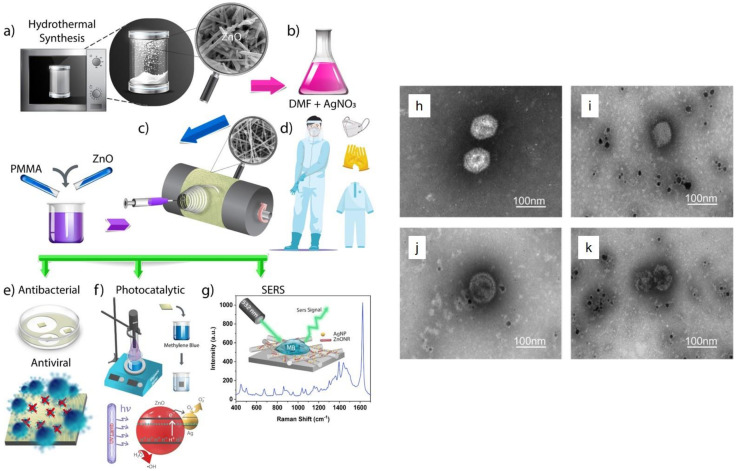
Schematic demonstration of the fabrication process of ZnO–Ag/PMMA nanofiber: (**a**) hydrothermal synthesis of ZnO nanorods. The inset image represents their morphology, (**b**) production of the electrospinning solution through addition of ZnO nanorods and PMMA to the AgNO_3_/DMF solution wherein Ag nanoparticles form via in situ reduction of the Ag salt, (**c**) creation of the ZnO–Ag/PMMA nanofibers through electrospinning, (**d**) the mats comprising the ZnO-Ag/PMMA nanofibers are employed as a protective clothing, and offer antimicrobial (**e**), photocatalytic (**f**), and sensing (**g**) properties. Reproduced with permission [144], Copyright 2021, American Chemical Society. TEM images show how the Ag nanoparticles adversely affect the Ad3 particles at different intervals. (**h**) Control sample in the absence of Ag nanoparticles; the samples treated with Ag nanoparticles (50 μg/mL) for 30 min (**i**), 90 min (**j**) and 120 min (**k**). As clearly observed, the capsid and the whole virus particle have been notably destructed in the presence of the nanoparticles. Reproduced with permission [158] Copyright 2013, Elsevier.

**Figure 9:**
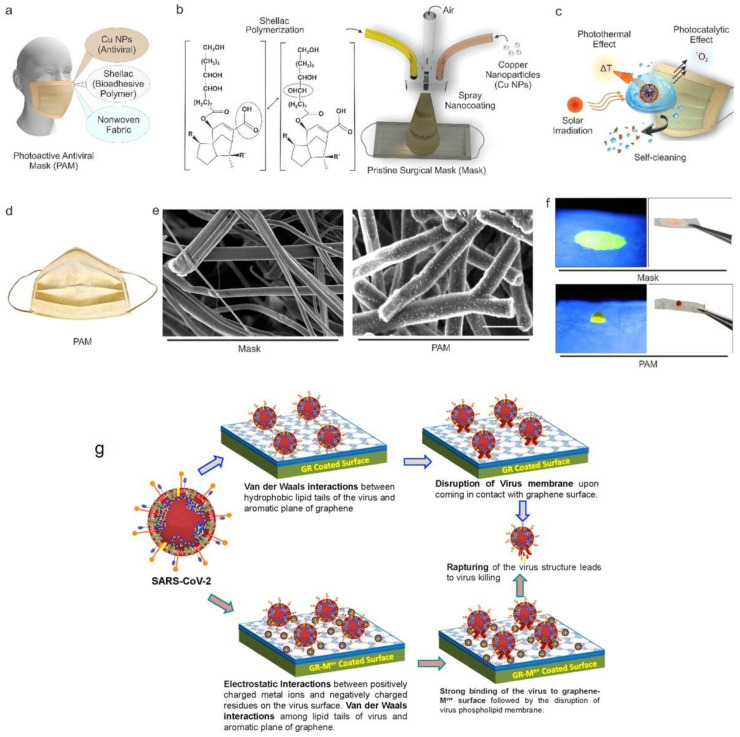
Schematic illustration of: (**a**) the antiviral nanohybrid coated surgical masks, (**b**) the dual-channel spray coating process on the nonwoven fibers of the surgical mask, whereby the Cu nanoparticle suspension and shellac are mixed and sprayed via the compressed N_2_ air channel, and (**c**) the solar light driven inactivation of the viruses available in the respiratory droplets. (**d**) Camera image of the photoactive antiviral mask (PAM). (**e**) SEM images show the morphology of propylene nonwoven fibers (**left**) in the commercial surgical mask and the nanohybrid-coated nonwoven fibers in the same mask (**right**). (Scale bar represents 10 μm). (**f**) Camera images of a dyed water droplet deposited on the uncoated mask and PAM after one hour. Reproduced with permission [154], Copyright 2021, American Chemical Society. (**g**) Schematic illustration of likely interactions between SARS-CoV-2 and graphene (GR) and GR nanohybrids comprising GR-metal ion, leading to virus inactivation on the coated surfaces. Reproduced with permission [167], Copyright 2020, Elsevier.

Polymer nanohybrids based on inclusion of antiviral agents in a polymeric nanomaterial and its later controlled release have been used for antiviral coatings. In this regard, metal oxides and nanoparticles are widely employed in construction of antiviral polymeric coatings. For instance, Karagoz et al. [144] synthesized nanohybrid nanofibers with antiviral and self-cleaning performance based on surface deposition of Ag nanoparticles and ZnO nanorods onto poly(methyl methacrylate) (PMMA) nanofibers, Figure 8a–c. Such nanohybrid nanofiber mats could offer excellent potential for protective clothing, Figure 8d, thanks to their promising antiviral performance against coronavirus and influenza virus, antibacterial activity for Gram-negative and -positive bacteria, and photocatalytic decomposition of organic pollutants, Figure 8e,f. Additionally, they provide a substrate allowing surface-enhanced Raman scattering (SERS) for the purpose of quantification of impurities mounted on the mat (fabric), Figure 8g. In another study, Tremiliosi et al. [145] synthesized Ag nanoparticle incorporated polycotton fabrics to reduce transmission of SARS-CoV-2. As will be discussed a bit later, Ag nanoparticles offer outstanding inactivation potential for pathogens and for this reason they are extensively employed in the textile industry. According to the results of this study, the proposed polymeric nanohybrid could optimally inactivate the SARS-CoV-2 virus (~100% in only 2 min), *E. coli and S. aureus bacteria*, and *Candida albicans* with no particular allergic reactions. Aligned with the use of natural polymers, alginate has been also used as a matrix for an antiviral nanohybrid system. Calcium alginate is well-known for its antiviral behavior against the enveloped double-stranded DNA herpes simplex virus type 1 [146]. To extend applicability of calcium alginate to non-enveloped double-stranded DNA viruses, carbon nanofibers (CNFs) have been added as a filler [147]. Recalling the promising antiviral activity of carbon nanomaterials, e.g., GO, against RNA and DNA viruses including pseudorabies virus and porcine epidemic diarrhea virus, the CNF/alginate benefits from a superior, synergetic antiviral performance originated from each constituent. It is believed that alginate’s negative charge is responsible of its antiviral activity [148] and hampers the decapsulation of the non-enveloped virus protein on the cell membrane’s surface [149]. Additionally, CNFs are also negatively charged and this charge is maximized due to their extremely large surface area [150,151,152,153]. This feature notably contributes to the overall surface charge of the nanohybrid and thereby notably inactivates the virus. In general, the nature-derived polymers with antimicrobial properties have been demanded considering their biocompatibility and abundance. In addition to cotton and alginate mentioned earlier, shellac has been also employed as a constituent of an antiviral nanohybrid. In this regard, Kumar et al. [154] developed a nanohybrid antiviral coating made of shellac/Cu nanoparticles that could be applied to nonwoven surgical masks, Figure 9a,b, to raise hydrophobicity, thereby avoiding adhesion of aqueous droplets. Additionally, the nanohybrid surface induced high photoactivity (a combination of photothermal and photocatalytic properties) that could provoke antimicrobial activity and enable self-sterilization and reusability of the masks. Exposed to solar light, the temperature of the mask promptly increases up to over 70 °C, thereby releasing a large number of free radicals that potentially damage the virus membrane, Figure 9c. Figure 9d,e show a general image of the photoactive antimicrobial mask (PAM) and the SEM images of its nonwoven fibers before and after coating with the antiviral nanohybrid. Figure 9f implies the hydrophobicity of the mask fibers after coating that could potentially prevent adhesion of the virus containing droplets on the mask surface.

An antiviral nanohybrid system comprising metal nanoparticles and metal oxides could be also applicable with respect to absorption of virus aerosols and inactivation of viruses. In this regard, Balagna et al. [155] developed an antiviral nanohybrid coating made of Ag nanoclusters/silica that could be deposited on metallic, glass, and cotton filters via co-sputtering. The as-made coatings showed a remarkable antiviral performance against respiratory viruses such as human rhinovirus and influenza A, while maintaining the filtering function. Such coatings can be suggested for modification of the masks and filters being used in gyms, hospitals, and anywhere that is exposed to the spread of viruses. Ag has been already recognized as an important antifungal and antibacterial material [156], and recently its antimicrobial effect has been extended to viruses [157] including adenovirus type 3 (Ad3) [158] and H1N1 influenza A virus [159]. Figure 8h–k shows how the Ad3 virus particles are damaged in the presence of Ag nanoparticles over a time period of 120 min. Further to the air filters coated with Ag nanoparticle/silica particles, Joe et al. [160] succeeded to verify that the as-developed nanohybrid system is able to show a promising antiviral effect against aerosolized bacteriophage MS2, while preserving the filtration efficiency.

Graphene, which is a two-dimensional (2D) one-atom-thick planar sheet of carbon atoms inter-*sp*^2^-bonded, is advantageous over classic nanomaterials in terms of extraordinary large surface area, carrier mobility, biocompatibility, and optical transparency [83]. Graphene exists in several forms of pristine graphene, graphene oxide (GO), and also reduced graphene oxide (rGO). Previously, the antibacterial potential of GO nanosheets has been validated through several relevant studies [161,162,163]. Additionally, it has been reported that graphene nanomaterials are able to block penetration and replication of RNA virus (coronavirus) and enveloped DNA virus (herpesvirus) in the target cells [164,165]. Synergistically, GO sheets alongside Ag nanoparticles form antimicrobial nanohybrids that can inactivate bacteria and viruses. In such nanohybrid systems, the GO sheets play a supportive/stabilizing role and hamper agglomeration of the nanoparticles and thus maintain the antimicrobial potential. As reported by de Faria et al. [161] the Ag nanoparticles mounted on GO sheets are spherical and as small as 7.5 nm, assuring their applicability for antiviral purpose. Thanks to the presence of GO sheets, the immobilized Ag nanoparticles are unable to displace and endanger the biocompatibility of the developed system. This feature minimizes the toxicity and the environmental hazards related to such nanoparticles. Additionally, the nanohybrid is notably water dispersible, possesses an extensive surface area, and offers outstanding antibacterial capacity at negligible amounts. Chen et al. [166] synthesized a nanohybrid comprised of Ag nanoparticles loaded GO sheets and investigated its antiviral potential against the feline coronavirus (FCoV; an enveloped virus) and infectious bursal disease virus (IBDV; a non-enveloped virus). As the authors report, the GO-Ag nanohybrid hampered 25% of the FCoV related infection and 23% of the IBDV caused infection. While, neat GO inhibited only 16% of the FCoV related infection and was ineffective against the infection arisen by IBDV. The as-developed GO-Ag nanohybrid can be potentially applied in construction of PPE to minimize virus transmission.

In general, GO nanomaterials exhibit a wide range of antiviral activities against DNA and RNA viruses, non-enveloped and enveloped viruses, and negative and positive sense viruses, thus offering promising potentials for generation of advanced antiviral coatings. In particular, knowing that the SAR-CoV-2 virus’ structure possesses a plethora of carboxyl groups and the virus hardly survives on the Cu surfaces, the coatings composed of GO/rGO-SO_3_ incorporated with Cu nanoparticles/ions could potentially inactivate the SARS-CoV-2 viruses. Other nanohybrids based on GO/rGO-SO_3_ doped with Ag, Ti, and Au could also be suggested for construction of antiviral coatings. As shown in Figure 9g, such materials optimally capture and destabilize the viral particles and thereby lower their survival chance when located on the antiviral coating [167]. In this regard, Van der Waals and electrostatic interactions are decisive in capturing the viruses and the sharp edges of the graphene plates disrupt the virus membrane, thereby killing the virus.

## 3. Conclusions and Future Perspective

The Covid-19 pandemic made us all encounter unpredicted challenges. Despite loss of many humans and irreversible damages to human society, this crisis had a second face of coin. It made us think how to prepare ourselves in the future for other likely virus pandemics. It taught us how to progress in science despite time and resource limitation and how to further consider the natural sources in dealing with such a crisis. Bioinspiration and employment of natural materials to cope with a natural crisis have advanced notably. Now, we mimic the structure and function of viruses to fight with them. The newly emerged vaccine systems that are in fact biohybrids in nanoscale are one of the mind-blowing achievements of human kind that was driven by surge of viral infections across the world. Now, we know that in the future we need multifunctional, bioinspired weapons against viruses. To meet this need, materials science and biology are coupled to generate a new generation of multifunctional and environmentally friendly nanohybrids that not only inactivate viruses, but also prevent their transmission and surface adhesion. Antiviral coatings are crucial to prevent the spread of viruses. On the other hand, the packaging materials that can show antimicrobial activity are highly demanded for disinfection of microorganisms. Furthermore, for the purpose of development of antiviral fabrics for PPE and household masks, antiviral nanohybrids will play a critical role.

In a broader perspective, we must seek advanced approaches guaranteeing health, but with consideration of the “One Health” concept, that stresses on the fact that the well-being of humans is tightly connected to that of other creatures and environment [1]. To tackle this intricate challenge, multidisciplinary researches involving scientists with complementing expertise are highly needed. The current crisis should be thought as a great opportunity to make us think about our globalized world and encourage us to take multi/interdisciplinary methodology more seriously. In this regard, transversal disciplines should be involved and the knowledge exchange and diversity in scientific point of views should be considered vital to create new, advanced scientific solutions. In this context, nanotechnology, particularly involving multifunctional nanohybrids, is a fertile area wherein researchers with different backgrounds can cooperate to address the complex problems. This frontline discipline is needed now more than ever to create new pillars toward resolving the current global public health issues, to weaponize us for the likely upcoming health challenges (e.g., infectious diseases), and to help us further consider sustainable scientific solutions.
